# Comparison of ECV measurements during equilibrium between IR- and SR-based Cardiac T_1_Mapping

**DOI:** 10.1186/1532-429X-16-S1-P53

**Published:** 2014-01-16

**Authors:** Kyungpyo Hong, Eugene G Kholmovski, Christopher J McGann, Ravi Ranjan, Daniel Kim

**Affiliations:** 1 UCAIR, Radiology, University of Utah, Salt Lake City, Utah, USA; 2 Division of Cardiology, Internal Medicine, University of Utah, Salt Lake City, Utah, USA

## Background

Cardiac T_1 _and extracellular volume fraction (ECV), derived from pre- and post-contrast cardiac and blood T_1 _measurements, are emerging imaging biomarkers of diffuse cardiac fibrosis. The most frequently used cardiac T_1 _mapping pulse sequence is MOLLI [1]. However, MOLLI is known to be sensitive to rapid heart rate and irregular rhythm, because it is based on inversion-recovery (IR) of magnetization preparation. In response, we developed an arrhythmia-insensitive-rapid (AIR) cardiac T1 mapping pulse sequence based on B_1_-insensitive saturation-recovery (SR) of magnetization preparation [2]. Our prior study [2] showed that AIR (scan time = 2-3 heart beats) is faster and yields more accurate cardiac T_1 _measurements than MOLLI (scan time = 17 heart beats). We sought to compare ECV measurements between SR-based AIR and IR-based MOLLI cardiac T_1 _mapping at 3T.

## Methods

Sixteen mongrel dogs with normal myocardium were imaged at 3T (Verio, Siemens). Cardiac T_1 _maps were acquired in a mid-ventricular short-axis plane using both AIR and MOLLI cardiac T_1 _mapping at baseline and during equilibrium of Gd-BOPTA (Multihance; 30 min after slow infusion at 0.002 mmol/kg/min). Note that equilibrium ensures identical concentration of Gd-BOPTA for a fair comparison of cardiac and blood T_1 _measured by two different pulse sequences. Both AIR and MOLLI acquisitions with b-SSFP readout were performed with the following relevant imaging parameters: spatial resolution = 1.4 × 1.4 × 7.0 mm, temporal resolution = 217 ms, flip angle = 35°, and SR time = 600 ms. The AIR acquisition was performed with "paired" consecutive phase-encoding steps in centric k-space ordering to minimize image artifacts due to eddy currents. Blood samples were drawn during MRI for hematocrit calculation. AIR and MOLLI cardiac T_1 _maps were manually segmented to calculate the myocardial and blood T_1 _values and subsequently ECV=(1-hematocrit)x(ΔR_1, myocardium_/ΔR_1, blood_), where R_1 _is T_1_^-1^, and Δ is the difference between post- and pre-contrast. Paired-wise t-test and Bland-Altman analyses were performed to compare the results.

## Results

**Figure 1:**
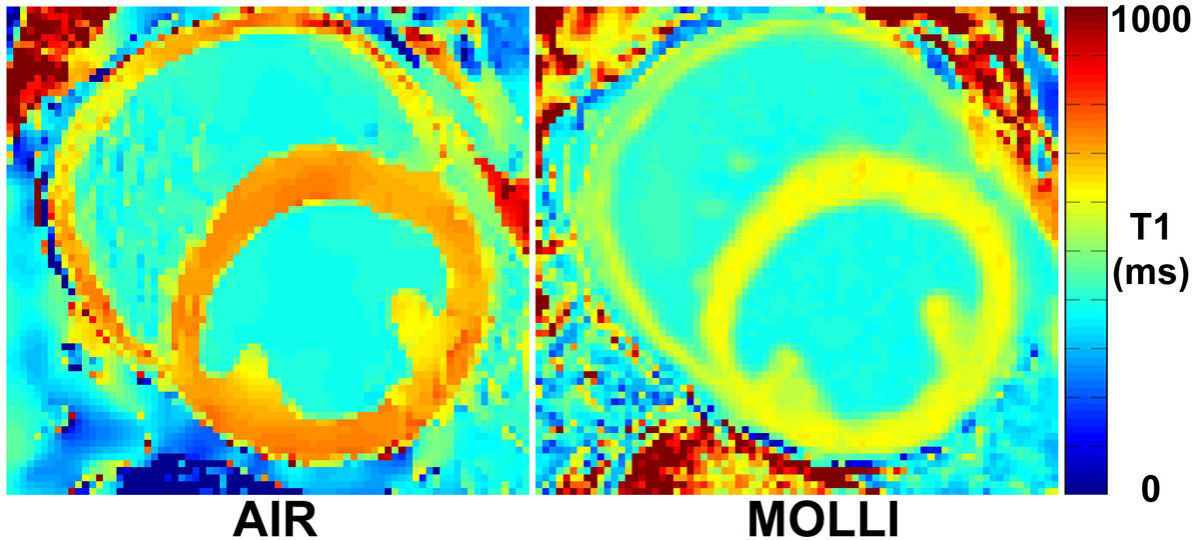
**Example SR-based AIR and IR-based MOLLI cardiac T_1 _maps of the same animal acquired during equilibrium**.

Figure 1 shows representative AIR and MOLLI cardiac T_1 _maps which exhibit similarly high image quality. In the 16 dogs studied (mean heart rate = 100 ± 19 BPM), compared with MOLLI, AIR yielded higher T_1 _measurements (mean difference = 185 ms; p < 0.0001) and lower ECV measurements (mean difference = -0.018; p < 0.0001).

## Conclusions

Our study suggests that MOLLI and AIR cardiac T_1 _mapping pulse sequences yield significantly different T_1 _and ECV measurements. ECV measurements derived from SR-based AIR and IR-based MOLLI cardiac T_1 _mapping pulse sequences may need to be adjusted for comparison

## Funding

Ben B. and Iris M. Margolis Foundation.

**Figure 2:**
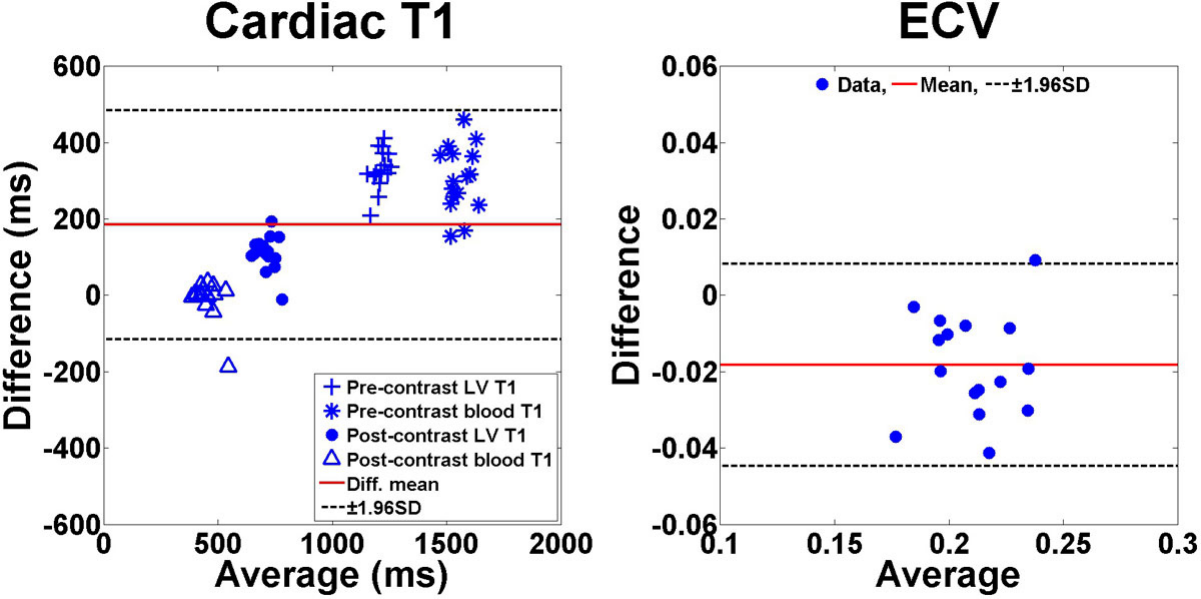
**Bland-Altman plots comparing (left) cardiac T_1 _and (right) ECV measurements derived from SR-based AIR and IR-based MOLLI cardiac T_1 _mapping**.
